# Veteran and Provider Satisfaction with a Home-Based Telerehabilitation Assessment for Wheelchair Seating and Mobility

**DOI:** 10.5195/ijt.2020.6341

**Published:** 2020-12-08

**Authors:** Kaila K. Ott, Richard M. Schein, Andi Saptono, Brad E. Dicianno, Mark R. Schmeler

**Affiliations:** 1 Department of Rehabilitation Science and Technology, School of Health and Rehabilitation Sciences, University of Pittsburgh, Pittsburgh, PA, USA; 2 Department of Health Information Management, School of Health and Rehabilitation Sciences, University of Pittsburgh, Pittsburgh, PA, USA; 3 Human Engineering Research Laboratories, VA Pittsburgh Healthcare System, Pittsburgh, PA, USA; 4 Department of Physical Medicine and Rehabilitation, University of Pittsburgh School Of Medicine, Pittsburgh, PA, USA

**Keywords:** Satisfaction, Service delivery, Telerehabilitation, Wheeled mobility

## Abstract

The objective of this project was to measure Veteran and provider satisfaction with a home-based telerehabilitation assessment for wheelchair seating and mobility. Forty-three Veterans were seen remotely at their place of residence by a provider, using a VA Video Connect synchronous videoconferencing system. Veteran and provider satisfaction were collected using the Telerehabilitation Questionnaire (TRQ). Mean individual TRQ scores for both Veterans and providers were significantly higher than the scale midpoint of 3.5. Veterans had higher scores than providers for five individual items on the TRQ. Higher scores by Veterans on the technology and quality and clarity of the video and audio likely correspond to the differences in environmental settings in which the visit occurred for the Veteran compared with the provider. High satisfaction scores with the telerehabilitation assessments are likely attributed to the positive working relationship between the provider and the rehabilitation technician, who provided in-person technical support to the Veteran in the home during the wheeled mobility evaluation. Overall, the results indicate a high level of Veteran and provider satisfaction using telerehabilitation for wheelchair seating and mobility evaluations.

Clients living in rural areas often face unique difficulties regarding the provision of healthcare services, such as lengthy travel time to medical facilities and lack of specialized providers and healthcare technology (Crandall & Coggan, 1994). The use of telehealth helps bridge the gap between individuals in need of specialized medical services and the location of such specialized care (Schmeler et al., 2009). “Telerehabilitation can be defined as the application of telecommunication, remote sensing and operation technologies, and computing technologies to assist with the provision of medical rehabilitation services at a distance” (Cooper et al., 2001). Overall, studies of telehealth services demonstrate very high levels of patient satisfaction, allowing more of a paradigm shift away from traditional in-person visits (Ramaswamy et al., 2020). The Department of Veteran Affairs (VA) telemedicine infrastructure is robust and saved Veterans 834,724 miles between 2005 and 2013, resulting in travel savings of 145 miles for each Veteran visit (Russo et al., 2016). Telerehabilitation helps to maximize Veteran health outcomes by connecting Veterans with providers in the most time effective manner (Gladden et al., 2015).

Telehealth specifically provides benefits for physical rehabilitation services, as defined by Lemaire et al., (2001) as: (1) decreased travel between rural communities and specialized urban health centers; (2) better clinical support in local communities; (3) improved access to specialized services; and (4) delivery of local health-care in rural communities. Physical Medicine and Rehabilitation services are often influenced by social and physical environmental factors; thus, providing telerehabilitation services in a naturalistic environment like the client's home, has much greater relevance, can identify critical barriers in the provision process of rehabilitative services, and increases the quality of healthcare services provided (McCue et al., 2010).

The World Report on Disability stated that telerehabilitation services produced similar or improved clinical outcomes compared with conventional in-person interventions (World Health Organization, 2011). Barlow et al. (2009) found that clients served by telerehabilitation and clients seen in-person were equally as likely to have their mobility goals met. Additionally, a study using the Functioning Everyday with a Wheelchair outcome tool showed that there were no significant differences between telerehabilitation and in-person services for seating and mobility evaluations, except for transportation (Schein et al., 2010a). Two separate studies demonstrated that clients are equally satisfied with telerehabilitation and in-person services for wheelchair assessments, using the Telerehabilitation Questionnaire (TRQ) and the Quebec User Evaluation of Satisfaction with Assistive Technology (QUEST) (Barlow et al., 2009; Schein et al., 2010b). Specifically, Schein et al. (2010b) demonstrated satisfaction with telerehabilitation services of individuals with mobility impairments in a private non-Veteran healthcare setting using the TRQ and that a scale midpoint of 3.5 was established as an appropriate cutoff to measure client satisfaction. Telehealth wheelchair seating and mobility assessments have the potential to continue to improve access in the provision of rehabilitation services; however, it is important to ensure high levels of engagement across all stakeholders to maintain optimal service delivery processes (Graham et al., 2020).

Three systematic reviews have been conducted on telehealth studies that evaluate client satisfaction and show that individuals are at least 80% satisfied with telehealth services, frequently reporting 100% satisfaction with the services received (Mair & Witten, 2000; Orlando et al., 2019; Williams et al., 2001). Kruse et al. (2017) and Donelan et al. (2019) explored the association between telehealth and client satisfaction and concluded that telehealth virtual visits are an important and useful option in clinical care and thus should be embraced and implemented due to its beneficial aspects, such as decreased client travel time, increase in access to care and communication, and improved client outcomes. Furthermore, Nguyen et al. (2020) reviewed both patient and provider satisfaction with telemedicine, revealing that it is important to consider factors that drive motivation for both stakeholders involved. Patients reported high levels of satisfaction with telemedicine (95-100%), whereas providers showed satisfaction when there was conditional support via the administration, self-involvement in the development process, and reliable and easy to use technology. Research conducted by Graham et al. (2020) showed that while consumers viewed telehealth wheelchair and seating assessments positively, the specialist assessors still had reservations. While research into the expansion of telehealth services is growing, there is still limited generalizability due to low sample sizes and limited context for defining and measuring client satisfaction (Orlando et al., 2019). Additionally, further research should be conducted on satisfaction from both the perspective of the client and the provider (Mair & Witten, 2000).

Much of the previous research conducted surrounding satisfaction of telerehabilitation services, focuses primarily on patients' perspectives, but this project wanted to identify the satisfaction experiences of both primary stakeholders, as well as how they compared to each other, specifically within the field of wheelchair seating and mobility. The objective of this project was to measure satisfaction with telerehabilitation services of both the Veterans and providers during a wheelchair seating and mobility assessment. The following hypotheses were identified:

1. Veterans' and providers' TRQ individual item responses will be significantly higher than the scale midpoint of 3.5, indicating satisfaction with the telerehabilitation assessment.
2. There will be a significant between-group differences in satisfaction with the telerehabilitation assessment for Veterans and providers, as measured by the TRQ individual items.

## MATERIALS AND METHODS

### INSTITUTIONAL REVIEW BOARD (IRB) APPROVAL

The VA Pittsburgh Healthcare System (VAPHS) IRB and University of Pittsburgh Human Research Protection Office were contacted prior to the start of this project to determine the research status of this project and if IRB approval was necessary. It was determined by both agencies that the project did not constitute research because the findings were designed and implemented for internal purposes; therefore, IRB review and approval were not needed. This project was determined to be a Quality Improvement project, and the VAPHS Quality Improvement Committee provided approval and permission to publish the results.

### SAMPLE

A screening process was implemented in the VAPHS Wheelchair, Seating, and Power Mobility Clinic in order to integrate telerehabilitation as a part of the routine clinical care for wheelchair seating and mobility assessments. Grenier (2018) provides a comprehensive overview of the development and implementation of the service delivery protocol used for this home-based telerehabilitation assessment for wheelchair seating and mobility. Consults for wheeled mobility evaluations are received and triaged by a wheelchair clinic therapist, known as the provider. According to the consult and chart review, the provider recommended the Veteran for a telerehabilitation assessment if: the Veteran's place of residence is within the perimeter of locations serviced by a rehabilitation technician (RT) for telerehabilitation wheelchair seating and mobility assessments, and the Veteran is medically and psychologically stable. The RT has specific training and skill sets in the application of rehabilitative and assistive technology to assist persons with disabilities in achieving greater independence and functional capability. The RT was part of the interdisciplinary team to assist in addressing problems related to wheelchair seating and mobility.

Further screening was performed by the RT through a phone assessment. Inclusion criteria were as follows: Veteran is alert and oriented; Veteran and/or caregiver is able to communicate needs and has the ability to comprehend clinical recommendations; Veteran can follow simple verbal, visual, or gestured requests independently or with the assistance of a caregiver; and Veteran and/or a caregiver is able and willing to participate in the telerehabilitation assessment. Veterans were excluded if: there were any concerns related to the safety and/or health of either the RT or the Veteran; there were any concerns that exceed the ability to meet the Veteran's clinical needs through a telerehabilitation encounter; the telerehabilitation team is unable to conduct a telehealth assessment at the Veteran's residence due to environmental factors, medical concerns, or technical limitations out of their control; and the Veteran's place of residence does not have reliable 4G/LTE service or internet connectivity. If the Veteran met all of the inclusion and exclusion criteria, they were scheduled for a wheelchair seating and mobility telerehabilitation assessment.

All types of residences were included for this project, including apartments, assisted living, and skilled nursing facilities. Project participants were seen for first-time mobility evaluations or repairs and modifications. Approximately 98% of participants were seen for an initial evaluation.

### INSTRUMENTATION

To conduct a telerehabilitation wheelchair seating and mobility assessment, a VA videoconferencing system, VA Video Connect (VVC), was used to provide synchronous communication (i.e., audio and visual) between the provider and the Veteran. The providers were physical therapists with specific expertise in wheelchair seating and mobility and had conducted other telerehabilitation assessments previously. The providers were located at the Wheelchair, Seating, and Power Mobility Clinic at the H.J. Heinz Campus in Pittsburgh, PA and the Veterans were located remotely at their place of residence with the RT. At the VA campus, the providers used a private office connected to their clinic equipped with a VA issued desktop computer and USB Web Camera. The VVC software with unique profiles for each telehealth provider was installed on the computer, which utilized encryption to ensure a private and secure connection between the provider and Veteran.

The RT traveled to the Veteran's place of residence for the appointment using a minivan to carry the necessary equipment. An Apple iPad Pro with the VVC application and different service provider mobile hotspot devices were used to wirelessly connect for each telerehabilitation encounter. The Qualtrics Offline Survey Application, a secure analytics software, was downloaded to the Apple iPad, allowing the RT to collect, store, and later analyze data collected from the Veterans during the evaluation. Providers' scores were collected via printed copies of the TRQ and later uploaded to the Qualtrics application upon collection by the RT. Furthermore, the RT traveled with demo wheelchair equipment provided by the local manufacturing representatives, allowing Veterans to test the equipment the provider recommended and ensure its appropriateness in meeting the Veterans' functional needs. The RT carried sanitation materials including gloves and sanitary wipes, a first aid kit, and tools for addressing any needed repairs, maintenance, or adjustments. Lastly, a project designated cell phone was used to contact the Veterans for appointment confirmation.

### MEASUREMENT TOOLS

#### DEMOGRAPHICS

An internal form was used to collect general demographics including age, gender, height, weight, and diagnosis contributing to the Veteran's need for a mobility device. Further information, including a Veteran's fall and pressure injury history and use of existing mobility assistive equipment, was collected to better understand the Veteran's current means of mobility and environmental factors, to help guide the clinical decision-making process.

#### TELEREHABILITATION QUESTIONNAIRE

Veteran and provider satisfaction were measured using the TRQ, a self-reported measurement tool. This short tool, although with minimal clinometric properties is the only tool developed to gather consumer satisfaction with telehealth wheelchair services (Malagodi et al., 1998; Schein et al., 2010b). The Veteran and provider completed the TRQ at the end of the telehealth assessment encounter. The TRQ contains seven items rated on a 6-point scale: 1 = completely disagree and 6 = completely agree. The survey's seven items are as follows:

1. I was comfortable being evaluated through this means.
2. The results of the evaluation through the tele-video conference would be as accurate as an evaluation being completed in person by a certified practitioner.
3. All areas of my lifestyle were considered with this process.
4. The technology did not interfere with the assessment.
5. The quality and clarity of the video and audio were acceptable.
6. Consulting with an expert clinician through tele-video conferencing saved me monetary expenses (i.e., travel time, gas, taking off work, family, etc.).
7. I would be willing to use this tele-video evaluation process again.

Team members met with providers prior to the start of this project and reviewed each TRQ item for appropriateness. All members mutually agreed that providers would view item 6 in terms of the provider's perspective and how tele-video conferencing would save their client monetary expenses.

### DATA ANALYSIS

IBM SPSS Statistics Version 24.0 was used to analyze the data. Alpha level was set at 0.05 for all analyses. To evaluate Veterans' and providers' satisfaction levels with the TRQ, one-sample *t*-tests were conducted to compare individual item means to the scale midpoint of 3.5. A Wilcoxon signed-rank test was performed to compare the TRQ individual item scores between the Veteran and provider for the telerehabilitation assessment.

## RESULTS

### DEMOGRAPHICS

**Figure 1:**
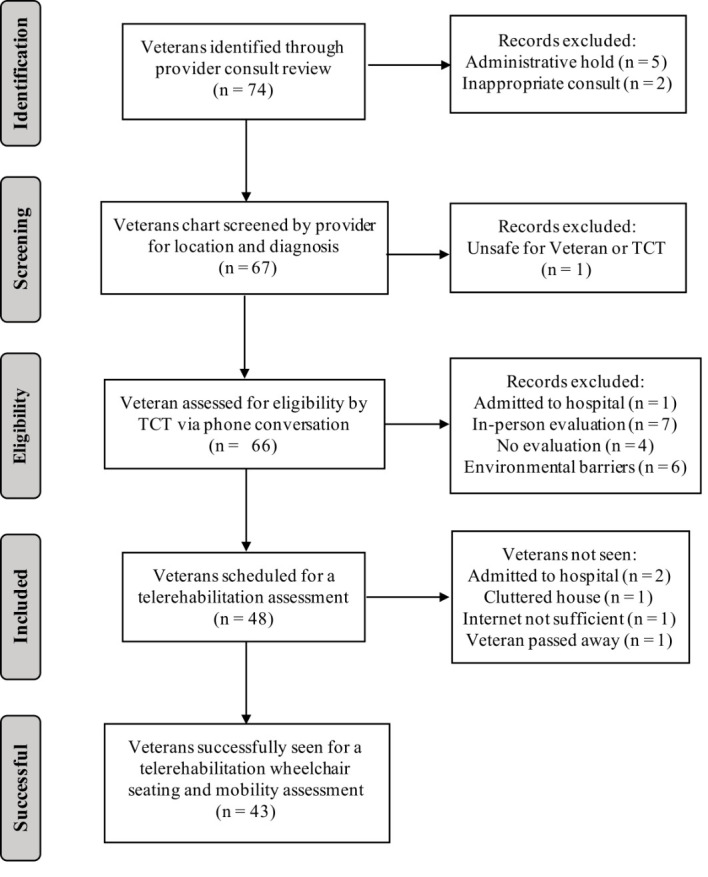
Flow Diagram for Veteran Screening

A total of 74 Veterans were screened for a telerehabilitation assessment between November 2017 and July 2018. Telerehabilitation assessments were successfully conducted for 43 Veterans. Figure 1 shows a flow diagram for Veteran screening. A breakdown of Veteran demographics can be seen in Table 1. The Veterans not seen via telerehabilitation were subsequently seen in-person for a wheelchair seating and mobility evaluation.

**Table 1:** Veteran Demographics

Demographics	N = 43
Age, *M±SD (min, max)*	81.7±9.1 (61,100)
Gender, *n(%)*	
Male	43 (100)
Ethnicity, *n(%)*	
White/Caucasian	39 (90.7)
Black/African American	4 (9.3)
Primary Diagnosis, *n(%)*	
Stroke	12 (27.9)
Other Neuromuscular or Congenital Disease	10 (23.3)
Cardiopulmonary Disease	7 (16.3)
Osteoarthritis	5 (11.6)
Parkinson Disease	3 (7.0)
Amputation	2 (4.7)
Spinal Stenosis	2 (4.7)
Spinal Cord Injury	2 (4.7)
Place of Residence, *n(%)*	
Community	34 (79.1)
Assisted Living	7 (16.3)
Skilled Nursing Facility	2 (4.7)
Mobility Assistive Equipment, *n(%)*	
Walker, Cane, Crutch	16 (37.2)
MWC^a^	17 (39.5)
PWC^b^	8 (18.6)
POV/Scooter	1 (2.3)
No Device	1 (2.3)

M = Mean; SD = Standard Deviation; MWC = manual wheelchair; PWC = power wheelchair; POV = power operated vehicle

a MWC = Manual wheelchair category includes transport, K0001, K0002, K0003, K0004, K0005, K0006, K0007, K0008, and K0009 manual wheelchairs

b PWC = Power Wheelchair category includes Group 1, Group 2, Group 3, Group 4, and Group 5 power wheelchairs

### TELEREHABILITATION QUESTIONNAIRE

All Veterans and providers who participated in the project responded to the TRQ. All mean scores, for both the Veterans and providers, were significantly higher than the scale midpoint of 3.5. A majority of Veterans reported that they ‘strongly agree' for each TRQ individual item, demonstrating high overall satisfaction with the telerehabilitation encounter (Table 2). Providers typically scored ‘mostly agree' or ‘strongly agree', on all TRQ items, except Items 4 and 5. Both items reflect about the telerehabilitation experience, whereas Item 4 specifically asks about whether the technology interfered with the assessment and Item 5 about the quality and clarity of the telerehabilitation encounter. A majority of provider scores were rated at ‘slightly agree' or higher (Table 3). While there is some variation in the providers' scores, the positive response from both the Veterans and providers indicates satisfaction with the telerehabilitation assessments.

**Table 2:** Veteran Satisfaction with Telerehabilitation Assessment

TRQ Item	Veteran Telerehabilitation Questionnaire Score, *n(%)*							One-sample *t*-test	
	1	2	3	4	5	6	*M* (SD)	95% CI	*p*^a^
1.Comfort	0 (0)	0 (0)	0 (0)	0 (0)	3 (7.0)	40 (93.0)	5.93 (0.26)	2.35-2.51	< 0.001
2.Accuracy	1 (2.3)	0 (0)	0 (0)	2 (4.7)	1 (2.3)	39 (90.7)	5.77 (0.87)	2.00-2.53	< 0.001
3.Lifestyle	0 (0)	0 (0)	0 (0)	1 (2.3)	3 (7.0)	39 (90.7)	5.88 (0.39)	2.26-2.50	< 0.001
4.Technology	1 (2.3)	0 (0)	0 (0)	2 (4.7)	1 (2.3)	39 (90.7)	5.77 (0.87)	2.00-2.53	< 0.001
5.Quality and Clarity	1 (2.3)	0 (0)	0 (0)	1 (2.3)	3 (7.0)	38 (88.4)	5.77 (.0.84)	2.01-2.53	< 0.001
6.Monetary Expenses	0 (0)	0 (0)	1 (2.3)	3 (7.0)	0 (0)	39 (90.7)	5.79 (0.68)	2.08-2.50	< 0.001
7.Repeated Use	1 (2.3)	0 (0)	0 (0)	1 (2.3)	1 (2.3)	40 (93.0)	5.81 (0.82)	2.06-2.57	< 0.001

*Note.* TRQ = Telerehabilitation Questionnaire; M = Mean; SD = Standard Deviation; CI = Confidence Interval

a p < 0.05

**Table 3:** Provider Satisfaction with Telerehabilitation Assessment

TRQ Item	Veteran Telerehabilitation Questionnaire Score, *n(%)*							One-sample *t*-test	
	1	2	3	4	5	6	*M* (SD)	95% CI	*p*^a^
1. Comfort	2 (4.7)	0 (0)	1 (2.3)	2 (4.7)	18 (41.9)	20 (46.5)	5.19 (1.16)	1.33-2.04	< 0.001
2. Accuracy	2 (4.7)	1 (2.3)	1 (2.3)	0 (0)	25 (58.1)	14 (32.6)	5.02 (1.19)	1.16-1.89	< 0.001
3. Lifestyle	1 (2.3)	0 (0)	1 (2.3)	0 (0)	14 (32.6)	27 (62.8)	5.49 (0.94)	1.70-2.28	< 0.001
4. Technology	3 (7.0)	1 (2.3)	0 (0)	6 (14.0)	19 (44.2)	14 (32.6)	4.84 (1.34)	0.92-1.75	< 0.001
5. Quality and Clarity	3 (7.0)	1 (2.3)	6 (14.0)	12 (27.9)	11 (25.6)	10 (23.3)	4.33 (1.41)	0.39-1.26	< 0.001
6. Monetary Expenses	0 (0)	0 (0)	1 (2.3)	1 (2.3)	5 (11.6)	36 (83.7)	5.77 (0.61)	2.08-2.46	< 0.001
7. Repeated Use	1 (2.3)	1 (2.3)	1 (2.3)	0 (0)	8 (18.6)	32 (74.4)	5.53 (1.08)	1.70-2.37	< 0.001

*Note.* TRQ = Telerehabilitation Questionnaire; M = Mean; SD = Standard Deviation; CI = Confidence Interval

a p < 0.05

A statistically significant difference was found between Veteran and provider scores on Items 1-5 of the TRQ. The providers consistently ranked aspects of the telerehabilitation encounter lower than the Veterans. Providers rated Item 4 (*M* = 4.84, *SD* = 1.34) and Item 5 (*M* = 4.33, *SD* = 1.41) much lower than the Veterans' scores for those items, Item 4 (*M* = 5.77, *SD* = 0.87) and Item 5 (*M* = 5.77, *SD* = 0.84). Item 6, regarding saved monetary expenses, *Z*(43) *= −0.16, p = 0.875,* and Item 7, regarding whether the individual would use telerehabilitation again, *Z*(43) *= −1.93, p = 0.053*, were not statistically different (Table 4).

**Table 4:** Veteran and Provider Wilcoxon Signed Rank Test

TRQ Item	Veteran TRQ	Provider TRQ	*Z*	*p*^a^
1. Comfort	5.93 (0.26)	5.19 (1.16)	−4.40	< 0.001
2. Accuracy	5.77 (0.87)	5.02 (1.19)	−3.82	< 0.001
3. Lifestyle	5.88 (0.39)	5.49 (0.94)	−3.13	0.002
4. Technology	5.77 (0.87)	4.84 (1.34)	−4.29	< 0.001
5. Quality and Clarity	5.77 (.0.84)	4.33 (1.41)	−4.79	< 0.001
6. Monetary Expenses	5.79 (0.68)	5.77 (0.61)	−0.16	0.875
7. Repeated Use	5.81 (0.82)	5.53 (1.08)	−1.93	0.053

*Note.* TRQ = Telerehabilitation Questionnaire

a p < 0.05; Sample Size = 43

## DISCUSSION

The growth of telehealth technologies helps to ameliorate concerns of Veterans with mobility limitations living in rural areas as well as address geographic and economic barriers in healthcare (McCue et al., 2010). This project specifically evaluated the satisfaction of both Veterans and providers during telehealth wheelchair seating and mobility evaluations. Previous research in the field of telehealth show consistent high levels of patient satisfaction, whereas the few studies that have evaluated provider satisfaction demonstrate satisfaction given certain criteria (Graham et al., 2020; Nguyen et al., 2020). This project hypothesized that similarly high levels of patient satisfaction would be measured; however, it was predicted that there would be differences in Veteran and provider satisfaction scores.

### VETERAN

The results of this project indicate that Veterans were satisfied with the telerehabilitation wheelchair seating and mobility assessments, consistent with previous research revealing high participant satisfaction with telehealth services (Donelan et al., 2019; Graham et al., 2020; Gustke et al., 2000; Mair & Witten, 2000; Nguyen et al., 2020; Ramaswamy et al., 2020; Schein et al., 2010b; Whitten & Love, 2005; Williams et al., 2001). A previous study by Gustke et al. (2000) revealed that patient satisfaction is rated high because the use of telehealth directly removes several problems associated with dissatisfaction in healthcare, such as appointment scheduling and travel time. Furthermore, for rural Veterans, high satisfaction may be due to a perceived increase in quality of care associated with the convenience of telehealth rather than individuals' true feelings of the services (Whitten & Love, 2005).

### PROVIDER

A similar study using the TRQ was conducted in the private medical sector, showing that all participant mean scores were significantly higher than the midpoint scale of 3.5, but Schein et al. (2010b) did report greater variation in Item 5, regarding the quality and clarity of the video and audio. While the current project did not detect that variation among Veteran scores, it did demonstrate similarities in the provider scores for Item 5, showing similarities to previous findings by Schein et al. (2010b). The home-based setting of this telerehabilitation project presented constraints related to the availability and strength of cellular signal or internet connectivity and moving the iPad around to give the provider the appropriate visual. While those factors were considered during the pre-screening process, fluctuations in quality and clarity of the video and audio throughout the assessment likely contributed to lower provider satisfaction scores on Item 5, in particular. Similarly, Whitten and Love (2005) found poor visual quality has been shown to directly impact the usefulness and perceived effectiveness of telehealth technology for providers.

Positive overall results and feedback from the providers might be attributed to the strong working relationship previously established between the provider and the RT. The rapport of the RT with the provider is crucial in the telerehabilitation experience for the provider, due to the knowledge, training, and experience required for wheelchair seating and mobility. The provider's confidence and trust in the RT's capabilities, impacts the ability to successfully conduct the assessment according to the provider's standards; thus, impacting the satisfaction levels recorded.

### VETERAN AND PROVIDER COMPARISON

This project detected important differences between the Veteran and provider TRQ scores. These discrepancies can be partly attributed to the differences in environmental settings between the Veteran and provider. During the telerehabilitation encounter, the provider is located in a private and quiet office with good lighting — an optimal setting for the Veteran to see and hear the provider clearly. In contrast, the provider must try to listen and see the Veteran in whatever the home telehealth setup may be. There were inherent differences in the settings of telehealth visits between the Veterans and providers; these became evident in their differences in satisfaction scores. Additionally, the provider may have higher expectations for the telerehabilitation system, given only preliminary experience using telerehabilitation for mobility assessments.

While previous studies evaluated satisfaction levels of patients using telehealth to receive healthcare services, there are very few studies that simultaneously researched the providers' satisfaction levels. Furthermore, there is no previous literature that assesses both stakeholders' satisfaction levels in the field of wheelchair seating and mobility. Based on previous systematic reviews, the research is clear that patients receiving care using telehealth modalities are highly satisfied with their services. The current project studied and identified the differences in satisfaction levels between patients and their providers. While both stakeholders' satisfaction responses showed overall positive experiences using telehealth, it is important to understand how the telehealth experience is different for each party for continued use and growth of this technology.

### PROJECT LIMITATIONS

Several limitations deserve discussion. First, this project was a 100% male Veteran sample, which is not representative of the gender distribution within the entire Veteran population. The Veteran Integrated Service Network covering Western Pennsylvania serves 4,501 female Veterans, representing only 6.4% of the total Veteran population in the area (U.S. Department of Veterans Affairs, 2016). The small percentage of female Veterans in the area, and specifically those with mobility limitations, made it difficult to include female Veterans. A second limitation was that the TRQ has not been psychometrically tested. However, the TRQ is one of the few tools available to capture patient satisfaction specific to the telerehabilitation service delivery process. While the TRQ outcome tool was developed and written to measure patient satisfaction, it was additionally used to measure provider satisfaction for the purpose of this project. The last limitation was that satisfaction for Veterans and providers were measured only at one time point. In future studies, satisfaction should be measured over time to continuously address and support Veterans' functional mobility.

### CONCLUSION

Telerehabilitation provides individuals with disabilities living in rural areas an effective and convenient way to receive specialized rehabilitative care. This project demonstrated that both male Veterans and providers were satisfied with a home-based telerehabilitation assessment for wheelchair seating and mobility when an RT was present in-person to support the process. This project is significant as it adapts wheelchair service delivery for a home-based telerehabilitation model, addresses the impact on a vulnerable population of Veterans with mobility limitations, and introduces a new healthcare team member, a rehabilitation technician, to assist in telerehabilitation methods. Telerehabilitation technology can help to improve access, quality, and continuity of care for Veterans with mobility limitations.
